# RELATIONSHIP OF VISUAL IMPAIRMENT AND POOR MOBILITY TO MORTALITY IN OLDER ADULTS

**DOI:** 10.1093/geroni/igae098.0090

**Published:** 2024-12-31

**Authors:** Atalie Thompson, Joseph Rigdon, Michael Miller, Rebecca Neiberg, Jeff Williamson, Stephen Kritchevsky

**Affiliations:** Wake Forest University School of Medicine, Winston-Salem, North Carolina, United States; Wake Forest University School of Medicine, Winston-Salem, North Carolina, United States; Wake Forest University School of Medicine, Winston-Salem, North Carolina, United States; Wake Forest University School of Medicine, Winston-Salem, North Carolina, United States; Atrium Health Wake Forest Baptist, Winston-Salem, North Carolina, United States; Wake Forest University School of Medicine, Wake Forest University School of Medicine, North Carolina, United States

## Abstract

Older adults with visual impairment (VI) have greater risk of mortality but the reasons are poorly understood. We have shown that older adults with VI are more likely to have poor mobility performance on the short physical performance battery (SPPB). In this analysis we examined whether VI predicted mortality over 10 years and if this was related to poor baseline mobility (SPPB< 9). We included 2457 older adults who completed three vision tests at the year 3 visit in the Health, Aging and Body Composition study. VI was defined by visual acuity < 20/40, log contrast sensitivity < 1.55 or stereoacuity >85. Cox proportional hazard models for mortality were adjusted for age, sex, black race, education, body mass index, smoking, depression, hypertension, heart disease, stroke, and diabetes. In separate models, VI (Hazard ratio (HR) 1.475, 95% CI (1.313-1.658), p< 0.0001) and SPPB< 9 (HR 1.469, 95% CI (1.233-1.75), p< 0.0001) each predicted mortality. When adding poor mobility (as a main effect and interaction with VI) and using unimpaired vision and mobility as the reference, those with only VI (HR 1.451, 95% CI(1.28-1.645), p< 0.0001) or only poor mobility (HR 1.495, 95% CI(1.117-2.0), p=0.0068) had similar HRs while those with both VI and poor mobility had an increased mortality risk (HR 1.943, 95% CI(1.562-2.418), p< 0.001), but the interaction was not significant (p=0.55). After accounting for major comorbidities, older adults with VI and poor mobility are at a greater risk of mortality. Future studies should consider if interventions to improve visual function and mobility improve survival in older adults.

